# Impact of the COVID-19 pandemic on lifestyle and mental state in patients with schizophrenia: A retrospective study

**DOI:** 10.1097/MD.0000000000032830

**Published:** 2023-02-03

**Authors:** Xiaoling Cheng, Xiaoling Huang, Xinghu Wu, Sulan Lin

**Affiliations:** a Department of Nursing, Quanzhou Third Hospital, Fujian, Quanzhou, China; b Department of Psychiatry, Hainan Province Anning Hospital, Haikou, Hainan Province, China.

**Keywords:** COVID-19, hospital management, nursing, schizophrenia

## Abstract

The corona virus disease 2019 pandemic’s movement restrictions have an effect on people’s lifestyles and mental health, and the most susceptible, such as persons with schizophrenia, are more subject to external influences. To analyze the lifestyle, anxiety, depression and stress status of inpatients with schizophrenia during corona virus disease 2019. A total of 205 patients with stable schizophrenia who were hospitalized during the epidemic period were selected. The general epidemiological information was recorded, and the depression screening scale (Patient Health Questionnaire-9), the Generalized Anxiety Disorder Scale-7, and the perceived stress scale were used to determine the mental status and stress level of the included patients. Restricted physical activity and perceptual bias might result in decreased food intake, higher body mass index, and increased psychotropic medicine dosages. According to Pearson correlation analysis, stress perception was positively connected with anxiety and depression scores. The anxiety score was positively associated with the depression score, indicating that stress influenced the patient’s emotional alterations. During the pandemic, the lifestyle and psychological load of people with schizophrenia may be impacted. Medical personnel should be alert to changes in anxiety, depression, and stress in patients with schizophrenia and take appropriate action.

## 1. Introduction

Since 2019, corona virus disease 2019 (COVID-19) has been raging worldwide. Not only has it had a huge impact on the national economy and policy, but people’s way of life has undergone tremendous changes as well.^[1]^ In addition to the general population, schizophrenia patients who have been hospitalized for a long time in special hospitals have received extensive attention from many psychiatrists because of the lack of information channels for the epidemic situation and the closed management of many hospitals. During the epidemic, family visits are prohibited, which aggravates negative emotions such as anxiety, depression and stress.^[2]^

The prevalence of schizophrenia is 21.4 per 100,000 person-years, with an average age of onset of 30.5 years.^[3]^ It is a chronic condition with a variety of symptoms such as: hallucinations, delusions, cognitive problems, withdrawal, indifference, and social dysfunction. These patients’ types of cognitive impairment makes these disorders one of the primary causes of disability, with substantial personal, societal, and economic effects.^[4,5]^ Conversely, patients with schizophrenia have a close relationship with their lifestyle, which includes food, physical activity, and social support. These characteristics have also been linked to the risk of various illnesses, including obesity, cardiovascular disease, and metabolic disorders.^[6,7]^ Education, socioeconomic position, and social isolation are all factors that contribute to these lifestyles.^[8]^

The isolation measures and mobility limitations used to contain the severe acute respiratory syndrome coronavirus 2 may have had a severe influence on that population’s way of life, which in turn may have affected the illness state of such individuals in the new environment of the COVID-19 pandemic. Therefore, the goal of this study was to examine how the prophylactic measures affected diet, exercise levels, and mentality of individuals with schizophrenia.

## 2. Materials and methods

This project is a retrospective study. Through online and offline questionnaires, data were collected from June 14, 2021 to June 15, 2022, from psychiatric hospitals in China that were affected by the epidemic. Participants responded to a survey but provided information about the study variables, involving 2 moments: restrictions on movement before and during the novel coronavirus pandemic. All participants input the data into the questionnaire under the supervision of the doctor.

### 2.1. Recruitment

Inclusion criteria: conforms to the International Classification of Diseases DSM-5 diagnostic criteria for schizophrenia^[9]^; does not restrict the dose and kind of antipsychotic medicines used; and all patients and guardians consented to participate in the study and gave informed permission. Exclusion criteria: patients with bipolar illness, mental retardation, or significant cognitive impairment were eliminated; and patients who were unable to complete the evaluation questionnaire effectively were removed midway.

### 2.2. Questionnaire

1. Socio-demographic information: gender, age, height, weight, amount of education, and occupational status.
2. The degree of patients’ physical activity was assessed using the International Physical Activity Questionnaire Short Form. This is a self-administered questionnaire with 7 items that gathered the pertinent data from respondents throughout the epidemic’s impacted time. Includes data on the frequency and duration of daily walking, sedentary activities, and vigorous or moderate activity.^[9]^
3. To evaluate the patients’ psychological states, the Generalized Anxiety Disorder Scale-7 (GAD-7), the depression screening scale (Patient Health Questionnaire-9 [PHQ-9]),^[11–13]^ and the Chinese version of the perceived stress scale (PSS)^[14,15]^ were employed.

GAD-7: The generalized anxiety inventory GAD-7 has 7 items. Each entry was graded 4, 3 = almost daily; 2 = more than 1 week; 1 = days away; 0 = not at all, the total score is the sum of the scores of the seven items, with a total score range of 0 to 21 points.

PHQ-9: The PHQ-9 contains a total of 9 items and asks for the answer that best matches the situation based on actual feelings during the last 2 weeks, making a judgment based on the first impression. Notes about options are as follows: none or very little time (within the past week, no more than 1 day when such situations appeared); days: fraction of the time (1–3 days had the condition in the past week); More than half of the time: quite a lot of time (within the past week, there have been such cases on about 4 days). Almost every day: most or all of the time (within the past week, there have been 5–7 days).

PSS measures how much stress people perceive themselves to be under as a result of an incident, which might lead to emotional or physical responses. The 5-point scale grading system was utilized, and assessment indications included the individuals’ sentiments, responses, and recognition. Four, 5, 6, 7, 9, 10, and 13 are reversal questions. Its standard “1” denotes never, “2” denotes practically never, “3” denotes sometimes, “4” denotes often, and “5” denotes always.

The internal consistency, as assessed by Cronbach alpha for PHQ9, GAD-7, and PSS was 0.824, 0.759, and 0.803, respectively.

### 2.3. Ethical statement

The essential guidelines outlined in the Helsinki Declaration were adhered to in this investigation. The characteristics of the study were appropriately disclosed to all participants, and completion of the survey was anonymous and voluntary.

### 2.4. Statistical analysis

Applying statistical analysis software Statistical Product Service Solutions 23.0, the Kolmogorov–Smirnov test was performed to determine if the normality hypothesis was met for each dependent variable. The *t* test was performed to compare the 2 groups, and the measurement data that followed the normal distribution were represented as (mean ± standard deviation). The 2 groups were compared using the *χ*^2^ test, and count results were reported as rate. Statistics were deemed significant at *P* = .05. The association between the depression, anxiety, and stress scales was assessed using the Pearson correlation coefficient, and the associated components of depression, anxiety, and stress were investigated using multiple linear regression analysis.

## 3. Results

The questionnaire survey based on the Internet and offline ended on June 15, 2022; the questionnaire was sent to 220 patients with schizophrenia and completed by 209 participants (95.0 %). After data validation, 205 respondents were eventually included in the study (4 questionnaires did not meet quality control requirements). The general characteristics of participants (mean ± standard deviation) are shown in Table 1.

**Table 1:** Demographic information of participants.

Age (yr)	59.18 ± 11.16
Body mass index (kg/m^2^)	22.42 ± 3.53
Female/Male (n)	73 (35.6)/132 (64.3)
Educational level	
Low	143 (69.7)
Intermediate	56 (27.3)
High	6 (2.9)

These values are expressed as the mean ± SD for age, body mass index, and as number (%) for gender and educational level.

In terms of nutritional consumption, 107 out of 205 patients (52.1%) had reduced dietary intake to varied degrees, showing that their appetite had diminished throughout the pandemic. Regarding physical exercise, the number of moderate activity and walking activity decreased from 54.7% before the epidemic to 38%, while the number of sits still increased from 93 (45.4%) to 127 (62%), implying that epidemic prevention and control measures can reduce the amount of exercise of patients. In addition, patients’ body mass index (BMI) increased generally during the outbreak (Table 2).

**Table 2:** Results of the impact on the BMI, physical activities and diet.

	Pre-COVID-19		Post-COVID-19		*P* value
Body mass index (kg/m^2^)	22.42 ± 3.53	24.08 ± 3.43		*P* < .001	
Dietary intake					
Increase	5 (2.4) 93 (45.3) 107 (52.1)				
Invariant					
Decrease					
Physical activity					
Intense activity	0	0			
Moderate activity	18 (8.8)	8 (3.9)		*P* < .01	
Walking activity	94 (45.9)	70 (34.1)			
Sit still	93 (45.4)	127 (62.0)			

BMI = body mass index, COVID-19 = corona virus disease 2019.

In respect of medication therapy, 115 (56%) of 205 patients reported not adjusting their drug therapy in light of the pre-pandemic circumstances, 90 patients reported increasing their dose, and no patients reported reducing their dosage (Table 3).

**Table 3:** Dosage of therapeutic drugs.

	Pre-COVID-19	Post-COVID-19
Medication intake		
Increase	–	90 (44.0)
Invariant	–	115 (56.0)
Decrease	–	0

COVID-19 = corona virus disease 2019.

The average PHQ-9 scale score revealed depression in 170 persons, with an 82.9% detection rate, including 14 people with severe depression, with a 6.8% detection rate. The GAD-7 scale revealed 127 persons with moderate or greater anxiety at a rate of 61.9%, including 7 people with moderate or severe anxiety at a rate of 3.4%. In general, the number of patients suffering from depression and anxiety increased compared with those before the epidemic prevention and control (*χ*^2^ test, *P* < .001) (Table 4).

**Table 4:** Depression and anxiety in patients with schizophrenia.

	Pre-COVID-19	Post-COVID-19	*P* value
PHQ-9			
No depression	86 (42.0)	35 (17.1)	*P* < .001
Mild depression	77 (37.6)	82 (40.0)	
Moderate depression	43 (21.0)	64 (31.2)	
Severe depression	7 (3.4)	24 (11.7)	
GAD-7			
No depression	55 (26.8)	35 (817.1)	*P* < .001
Mild depression	85 (41.5)	72 (35.1)	
Moderate depression	57 (27.8)	91 (44.4)	
Severe depression	8 (3.9)	7 (3.4)	

COVID-19 = corona virus disease 2019, GAD-7 = Generalized Anxiety Disorder Scale-7, PHQ-9 = Patient Health Questionnaire-9.

The average PSS scale score was 157 persons with stress perception and the detection rate was 76.5%; 9 people felt apparent pressure and the detection rate was 4.3%. Statistics show a difference in the number of patients before and after the pandemic (*χ*^2^ test, *P* < .001) (Table 5).

**Table 5:** Pressure situation in patients with schizophrenia.

	Pre-COVID-19	Post-COVID-19	*P* value
PSS			
Low perceived pressure	95 (46.3)	49 (23.9)	*P* < .001
Moderate perceived stress	67 (36.7)	92 (44.9)	
High perceived stress	37 (18.0)	56 (27.3)	
Extremely high perceived stress	6 (3.0)	8 (3.9)	

COVID-19 = corona virus disease 2019, PSS = perceived stress scale.

Stress perception was positively correlated with anxiety score and depression score, respectively, according to Pearson correlation analysis (*P* < .01); there was also a positive correlation between anxiety score and depression index (*P* < .01), indicating that patients’ emotional changes were influenced by stress perception (Table 6).

**Table 6:** Correlation analysis between stress, depression and anxiety.

	PHQ-9	GAD-7	PSS
PHQ-9	1	0.617*	0.417*
GAD-7	0.617*	1	0.563*
PSS	0.417*	0.563*	1

GAD-7 = Generalized Anxiety Disorder Scale-7, PHQ-9 = Patient Health Questionnaire-9, PSS = perceived stress scale.

Pearson correlation analysis; * Significant correlation at 0.01 level (bilateral).

## 4. Discussion

COVID-19 has become one of the public security events of the “global pandemic.” Due to the rapid spread and uncertainty of the virus, it directly increases the psychological burden of people.^[16,17]^ After COVID-19 infection, the general population with stable clinical symptoms also showed significant stress response.^[18]^ In 2005, relevant researchers found that under the “SARS” epidemic situation, there were mental health problems of varying degrees induced by psychiatric patients.^[19]^ The focus of this research was to look at how people with schizophrenia changed their lifestyle and levels of anxiety and depression during the COVID-19 pandemic lockdown. So far, there have been little research on food, exercise, anxiety, depression, and stress levels in this population. The survey employed in this investigation had a 95% response rate, which is comparable to other recent studies in similar patient populations.

### 4.1. Diet, exercise and BMI

Patients with schizophrenia have been shown to benefit from exercise, but in everyday life, these individuals are often sedentary^[19]^ or tend to exercise at a lower or moderate intensity than the general population.^[20]^ During the epidemic, many hospitals adopted closed management, limiting patient activities and reducing unnecessary visits.^[21]^ As a result, the amount of activity in patients with schizophrenia has decreased to varying degrees, accompanied by increased BMI and decreased food intake. In addition, due to the lack of ways for patients to receive information, the perception of lifestyle changes will also affect appetite to some extent.

### 4.2. Medication

In terms of pharmacological therapy, 56% of participants did not alter their regimen compared to before the pandemic. Patients reported adjusting the dosage 44% of the time. The findings of a research done in the Spanish population revealed that,^[22]^ in comparison to the general population, those with mental illnesses had higher levels of anxiety and sadness during the sitting months. This finding may be connected to the increased pharmacological therapy we noticed.

### 4.3. Anxiety, depression and stress perception

Participants’ scores on the 2 PHQ-9, GAD-7 subscales for anxiety and depression significantly increased under the COVID-19 pandemic movement limitations compared to their pre-pandemic levels. During the pandemic, 82.9% of patients had mild, moderate, or severe depression symptoms, compared to 58% prior. The number of individuals exhibiting anxiety symptoms increased by 10.2% during the lockdown, which was also a similar increase to the number of depressive episodes. Additionally, these findings concur with those of a cross-sectional study carried out in China during the COVID-19 epidemic.^[23]^

The results of a Pearson correlation analysis revealed that stress perception was positively correlated with anxiety score and depression score, respectively. Since anxiety score and depression index are positively correlated, it is clear that patients’ emotional changes are influenced by their pressure levels. Analysis of the causes: hospitalized schizophrenics rely heavily on television news, newspapers, magazines, and other public media as their only source of external knowledge. Additionally, these patients suffer from cognitive impairments that make it difficult for them to absorb and interpret epidemic information, which can easily trigger unpleasant feelings. As a consequence, the information gathering about the epidemic condition steadily worsens. In order to prevent their anxiety, despair, tension, and other bad feelings, schizophrenia patients must suitably restrict their knowledge intake about the pandemic condition throughout their daily rehabilitation.

There are some drawbacks to this study, including memory bias. Furthermore, because we recruit from various medical institutes in China, the samples are not homogenous. Finally, we solely gathered data on changes in the dose of medications taken during the pandemic, without considering the potential influence of drug type. The study was retrospective based on a Chinese population, and to improve its internal validity, we recruited subjects in a randomized manner, collected data using standardized questionnaires and scales, consulted a statistical expert to guarantee the use of suitable statistical methods, and as far as we could expand the sample size. Nevertheless, due to the large variations in the cultural backgrounds, diagnosis and treatment environments and social environments of populations in different regions, the results of our study can only be used as a reference, and the corresponding studies in a specific region are more highly informative. We also expect a large number of multicenter, multi-region prospective studies to provide more reliable conclusions.

## 5. Conclusion

To summarize, the epidemic era can have a significant influence on the lifestyle and psychological burden of schizophrenia patients. As a result, psychiatric medical care personnel must pay attention to changes in anxiety, depression, and stress levels in patients with schizophrenia, increase health education and behavioral guidance on the patient’s new coronavirus disease knowledge and personal protection, and employ psychological interventions.

## Author contributions

**Conceptualization:** Xiaoling Cheng, Xiaoling Huang, Xinghu Wu.

**Data curation:** Xiaoling Cheng, Xiaoling Huang.

**Formal analysis:** Xiaoling Cheng, Xiaoling Huang.

**Investigation:** Xiaoling Cheng, Xiaoling Huang, Xinghu Wu.

**Methodology:** Xinghu Wu.

**Supervision:** Xinghu Wu, Sulan Lin.

**Validation:** Sulan Lin.

**Writing – original draft:** Xiaoling Cheng, Xiaoling Huang.

**Writing – review & editing:** Xinghu Wu, Sulan Lin.

## References

[1] Giuntella O Hyde K Saccardo S. Lifestyle and mental health disruptions during COVID-19. Proc Natl Acad Sci U S A. 2021;118. 10.1073/pnas.2016632118 PMC7936339 33571107

[2] Zhand N Joober R. Implications of the COVID-19 pandemic for patients with schizophrenia spectrum disorders: narrative review. BJPsych Open. 2021;7:e35. 33431109 10.1192/bjo.2020.157 PMC7804069

[3] Jongsma HE Gayer-Anderson C Lasalvia A. Treated incidence of psychotic disorders in the multinational EU-GEI study. JAMA Psychiat. 2018;75:36–46. 10.1001/jamapsychiatry.2017.3554 PMC5833538 29214289

[4] Zeidler J Slawik L Fleischmann J. The costs of schizophrenia and predictors of hospitalisation from the statutory health insurance perspective. Health Econ Rev. 2012;2:9. 22828440 10.1186/2191-1991-2-9 PMC3464783

[5] Global, regional, and national incidence, prevalence, and years lived with disability for 301 acute and chronic diseases and injuries in 188 countries, 1990-2013: a systematic analysis for the Global Burden of Disease Study 2013. Lancet. 2015;386:743–800. 26063472 10.1016/S0140-6736(15)60692-4 PMC4561509

[6] Vancampfort D Knapen J Probst M. Considering a frame of reference for physical activity research related to the cardiometabolic risk profile in schizophrenia. Psychiatry Res. 2010;177:271–9. 20406713 10.1016/j.psychres.2010.03.011

[7] Ratliff JC Palmese LB Reutenauer EL. The effect of dietary and physical activity pattern on metabolic profile in individuals with schizophrenia: a cross-sectional study. Compr Psychiatry. 2012;53:1028–33. 22425530 10.1016/j.comppsych.2012.02.003 PMC3380150

[8] Heald A Pendlebury J Anderson S. Lifestyle factors and the metabolic syndrome in schizophrenia: a cross-sectional study. Ann Gen Psychiatr. 2017;16:12. 10.1186/s12991-017-0134-6 PMC5310063 28289436

[9] DSM-5: diagnosis of mental disorders. Lancet. 2010;376:390. 10.1016/S0140-6736(10)61204-4 20692512

[10] Faulkner G Cohn T Remington G. Validation of a physical activity assessment tool for individuals with schizophrenia. Schizophr Res. 2006;82:225–31. 16360305 10.1016/j.schres.2005.10.020

[11] Zimmerman M. Using the 9-item patient health questionnaire to screen for and monitor depression. JAMA. 2019;322:2125–6. 31626276 10.1001/jama.2019.15883

[12] Negeri ZF Levis B Sun Y. Accuracy of the Patient Health Questionnaire-9 for screening to detect major depression: updated systematic review and individual participant data meta-analysis. BMJ. 2021;375:n2183. 34610915 10.1136/bmj.n2183 PMC8491108

[13] Bone C Simmonds-Buckley M Thwaites R. Dynamic prediction of psychological treatment outcomes: development and validation of a prediction model using routinely collected symptom data. Lancet Digit Health. 2021;3:e231–40. 33766287 10.1016/S2589-7500(21)00018-2

[14] Maswadi N Khader YS Abu SA. Perceived stress among resident doctors in Jordanian teaching hospitals: cross-sectional study. JMIR Public Health Surveill. 2019;5:e14238. 31579024 10.2196/14238 PMC6777282

[15] Khalili R Sirati NM Ebadi A. Validity and reliability of the Cohen 10-item perceived stress scale in patients with chronic headache: Persian version. Asian J Psychiatr. 2017;26:136–40. 28483077 10.1016/j.ajp.2017.01.010

[16] Anda S Budd EL Halvorson S. Effects of a health education intervention for COVID-19 prevention in Latinx communities: a cluster-randomized controlled trial. Am J Public Health. 2022;112:S923–7. 36446063 10.2105/AJPH.2022.307129 PMC9707712

[17] ElTohamy A Wang JJ Chen JA. Association between college course delivery model and rates of psychological distress during the COVID-19 pandemic. JAMA Netw Open. 2022;5:e2244270e2244270. 36449292 10.1001/jamanetworkopen.2022.44270 PMC9713601

[18] Salanti G Peter N Tonia T. The impact of the COVID-19 pandemic and associated control measures on the mental health of the general population: a systematic review and dose-response meta-analysis. Ann Intern Med. 2022;175:1560–71. 36252247 10.7326/M22-1507 PMC9579966

[19] Lau JT Yang X Tsui HY. Positive mental health-related impacts of the SARS epidemic on the general public in Hong Kong and their associations with other negative impacts. J Infect. 2006;53:114–24. 16343636 10.1016/j.jinf.2005.10.019 PMC7132442

[20] Ohi K Kataoka Y Shimada T. Meta-analysis of physical activity and effects of social function and quality of life on the physical activity in patients with schizophrenia. Eur Arch Psychiatry Clin Neurosci. 2019;269:517–27. 29789938 10.1007/s00406-018-0903-5

[21] Bivia-Roig G Soldevila-Matias P Haro G. The impact of the COVID-19 pandemic on the lifestyles and levels of anxiety and depression of patients with schizophrenia: a retrospective observational study. Healthcare (Basel). 2022;10. 10.3390/healthcare10010128 PMC8776060 35052292

[22] Sole B Verdolini N Amoretti S. Effects of the COVID-19 pandemic and lockdown in Spain: comparison between community controls and patients with a psychiatric disorder. Preliminary results from the BRIS-MHC STUDY. J Affect Disord. 2021;281:13–23. 33279864 10.1016/j.jad.2020.11.099 PMC7683299

[23] Li W Zhao N Yan X. The prevalence of depressive and anxiety symptoms and their associations with quality of life among clinically stable older patients with psychiatric disorders during the COVID-19 pandemic. Transl Psychiatry. 2021;11:75. 33500389 10.1038/s41398-021-01196-y PMC7835649

